# The pH Effects on SARS-CoV and SARS-CoV-2 Spike Proteins in the Process of Binding to hACE2

**DOI:** 10.3390/pathogens11020238

**Published:** 2022-02-11

**Authors:** Yixin Xie, Wenhan Guo, Alan Lopez-Hernadez, Shaolei Teng, Lin Li

**Affiliations:** 1 Computational Science Program, University of Texas at El Paso, El Paso, TX 79912, USA; yxie4@miners.utep.edu (Y.X.); wguo1@miners.utep.edu (W.G.); aelopezhern@miners.utep.edu (A.L.-H.); 2 Department of Biology, Howard University, Washington, DC 20059, USA; shaolei.teng@howard.edu; 3 Department of Physics, University of Texas at El Paso, El Paso, TX 79912, USA

**Keywords:** SARS-CoV, SARS-CoV-2, COVID-19, electrostatic features, Angiotensin-Converting Enzyme 2, hACE2, spike protein, pH dependence, binding energy, folding energy, hydrogen bonds

## Abstract

COVID-19 has been threatening human health since the late 2019, and has a significant impact on human health and economy. Understanding SARS-CoV-2 and other coronaviruses is important to develop effective treatments for COVID-19 and other coronavirus-caused diseases. In this work, we applied multi-scale computational approaches to study the electrostatic features of spike (S) proteins for SARS-CoV and SARS-CoV-2. From our results, we found that SARS-CoV and SARS-CoV-2 have similar charge distributions and electrostatic features when binding with the human angiotensin-converting enzyme 2 (hACE2). Energy pH-dependence calculations revealed that the complex structures of hACE2 and the S proteins of SARS-CoV/SARS-CoV-2 are stable at pH values ranging from 7.5 to 9. Three independent 100 ns molecular dynamics (MD) simulations were performed using NAMD to investigate the hydrogen bonds between S proteins RBD and hACE2 RBD. From MD simulations, we found that SARS-CoV-2 forms 19 pairs (average of three simulations) of hydrogen bonds with high occupancy (>50%) to hACE2, compared to 16 pairs between SARS-CoV and hACE2. Additionally, SARS-CoV viruses prefer sticking to the same hydrogen bond pairs, while SARS-CoV-2 tends to have a larger range of selections on hydrogen bonds acceptors. We also labelled key residues involved in forming the top five hydrogen bonds that were found in all three independent 100 ns simulations. This identification is important to potential drug designs for COVID-19 treatments. Our work will shed the light on current and future coronavirus-caused diseases.

## 1. Introduction

The ongoing COVID-19 pandemic is changing human society significantly and causing both economic and social consequences all over the world [1]. Coronaviruses are named for their crown-like spikes on their surface, and they are commonly found in many mammal species [2]. Human coronaviruses were firstly identified in the mid-1960s. There are four main sub-groupings of coronaviruses, known as alpha, beta, gamma, and delta [3]. Among all the coronaviruses, there are seven known types of coronaviruses that can infect human beings. People around the world are commonly infected by human coronaviruses 229E, NL63, OC43, and HKU1 [4,5]. Additionally, some coronaviruses that infect animals are able to evolve and infect humans, among which the three recent cases are SARS-CoV-2, SARS-CoV, and MERS-CoV [6]. The SARS-CoV-2 virus is the novel coronavirus that causes coronavirus disease 2019, or COVID-19. Other than COVID-19, coronaviruses have caused several pandemics before, including severe acute respiratory syndrome (SARS), which was caused by SARS-CoV, and the Middle East respiratory syndrome (MERS), which was caused by MERS-CoV. To end the current pandemic soon and be prepared for the future similar challenges for human society, it is essential to understand the binding mechanisms of SARS-CoV-2 infecting human cells. This is achievable by studying the stabilities of SARS-CoV-2 at different pH conditions and identifying the key residues that play significant roles in the binding processes.

Coronaviruses contain membrane glycoprotein (M), nucleocapsid protein (N), spike protein (S), envelope protein (E), and an RNA single chain [7]. For all enveloped viruses, one of the most important steps during the binding process is membrane fusion, which allows viruses to infiltrate host cells [8]. For coronaviruses, the fusion protein is the S protein that leads the binding process to attack human cells through the host cell receptor angiotensin-converting enzyme 2 (hACE2) [9]. Human hACE2 (hACE2) is an enzyme located widely in the human body, including the lungs, kidneys, adipose tissue, central nervous system, and cardiovascular system [9,10,11], and it has multiple essential functions such as the regulation of amino acid transport in the kidney controlling the blood pressure, and viral receptors including both SARS-CoV-2 and SARS-CoV [11]. Since it is of extreme importance to human health, numerous research groups have been or are currently working on S proteins and hACE2 using various approaches.

The traditional process of the de novo drug design is a challenging task that consumes resources and time significantly. With the fast developments of computing technology, computational methods have been widely used in drug-related research [12], including protein–protein interactions [13,14], MD simulations [15], coarse-grained models [16], pH dependence of protein–protein interactions [17,18,19,20], etc. Our previous studies have applied multi-scale computational methods to study several pathogens [21,22,23,24,25] including the SARS-CoV-2 viruses [26,27], which revealed some mechanisms of the SARS-CoV-2 S protein. Additionally, many other research groups have made successful progress to understand the SARS-CoV-2 using computational methods [28,29].

In this work, we firstly calculated the electrostatic potentials on the surface of S proteins from both SARS-CoV and SARS-CoV-2, followed by the electric field line comparison between SARS-CoV and SARS-CoV-2 when they bind to hACE2. We found that these two viruses have similar pH responses: the pH-dependence of folding energies for S protein receptor binding domains (RBDs) demonstrated that both the S protein RBDs of these two viruses are at the most stable status when pH values ranging from 6 to 9. The pHvdependence of binding energies for S protein RBDs and hACE2 RBD showed that the complex structures of the two viruses are at the most stable status at pH values ranging from 7.5 to 10.5. Therefore, pH 7.5 to 9 is the best condition for both SARS-CoV and SARS-CoV-2 to perform their functions of binding with hACE2. Additionally, we analyzed the trajectories from three independent 100ns MD simulations for each complex structure using NAMD [30] and identified essential hydrogen bonds with the involved key residues using VMD [31]. This work mainly focuses on the analyses of hydrogen bonds. Our previous studies discussed ionic interactions in detail [26,27]. From the MD simulations we found that SARS-CoV-2 forms average 19 pairs hydrogen bonds with high occupancy (>50%) to hACE2, compared to 16 pairs between SARS-CoV and hACE2. Additionally, SARS-CoV tends to stick to same hydrogen bond pairs, while SARS-CoV-2 tends to have a larger range of selections on hydrogen bonds acceptors. We also labelled key residues involved in forming the top five hydrogen bonds that were found in all three independent 100 ns simulations. This identification is important to potential drug designs for COVID-19 treatments. Our work will shed light on current and future coronavirus-caused diseases.

## 2. Results and Discussions

First of all, the electrostatic features of SARS-CoV and SARS-CoV-2 S proteins were investigated, including electrostatic potential and electric field lines. Secondly, the relative binding energies of complex structures and folding energies of S proteins at different pH values were analyzed. Finally, the hydrogen bonds and related key residues in each complex structure were obtained using MD simulations.

### 2.1. S Protein Trimer Structure

**Figure 1:**
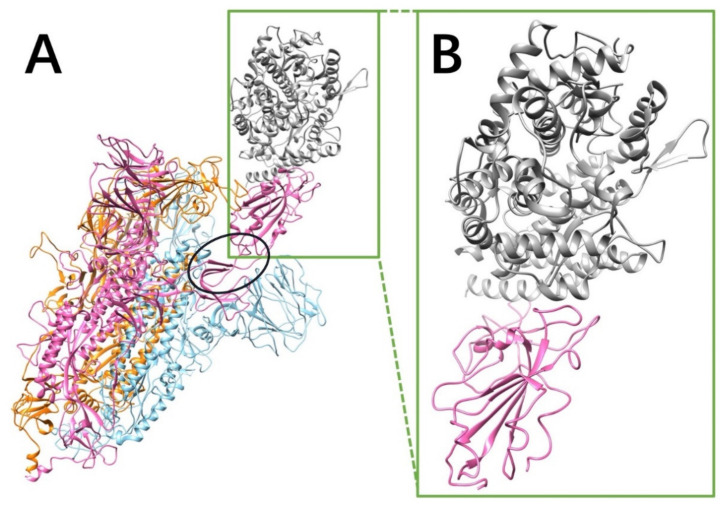
SARS-CoV S protein structure. Only the SARS-CoV S protein structure is illustrated in this figure, because SARS-CoV and SARS-CoV-2 S proteins are very similar (the RMSD between two S protein RBDs is 0.973 Å). (**A**) The S protein is a homotrimer (orange, blue, pink), of which one chain (pink) flips out when it binds to hACE2 (gray). The hinge connecting the RBD and the other part of S protein is shown in a black circle; (**B**) the closeup view of binding domains when S protein RBD (pink) binds to hACE2 RBD (gray).

The RMSD between the S proteins of SARS-CoV and SARS-CoV-2 is 0.973 Å, showing that the S proteins of SARS-CoV and SARS-CoV-2 are very similar. The S proteins of SARS-CoV and SARS-CoV-2 are both homotrimers. Each monomer contains an RBD, which connects the other part of the monomer via a hinge composed by two flexible loops (as shown in the black circle of Figure 1A). The RBD is in closed configuration when there is no hACE2 binds to the S protein. When binding to hACE2, the RBD of one monomer flips out as open configuration, and it binds to the RBD of hACE2.

### 2.2. Electrostatic Potential on Surfaces

**Figure 2:**
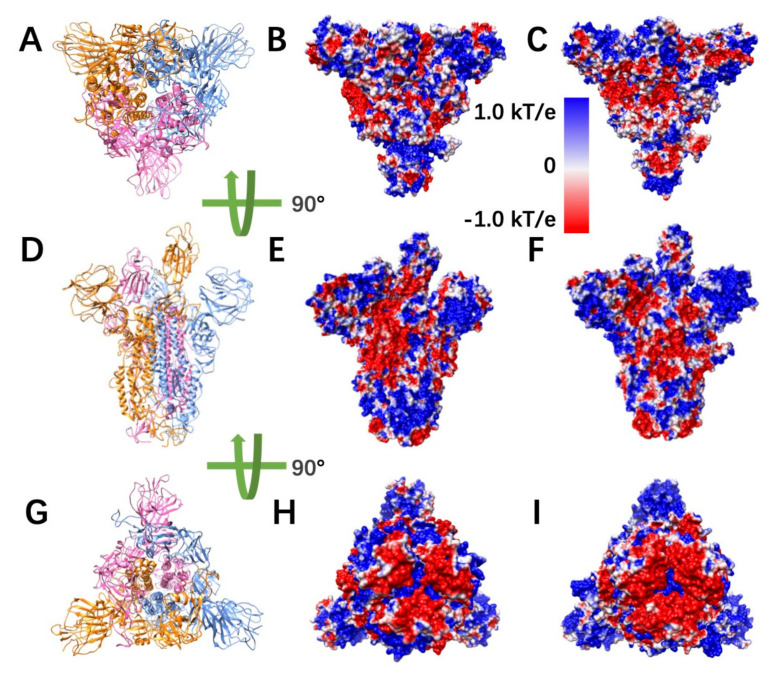
Electrostatic potential on surfaces of SARS-CoV and SARS-CoV-2 S proteins. (**A**) Top view of S protein structure; (**B**,**C**) top views of electrostatic potential on surfaces of SARS-CoV and SARS-CoV-2 S protein, respectively; (**D**) front view of S protein structure; (**E**,**F**) front views of electrostatic potential on surfaces of SARS-CoV and SARS-CoV-2 S protein, respectively; (**G**) bottom view of S protein structure; (**H**,**I**) bottom views of electrostatic potential on surfaces of SARS-CoV and SARS-CoV-2 S protein, respectively. Negatively and positively charged areas are colored in red and blue, respectively, with the color scale from −1.0 to 1.0 kT/e.

To study the electrostatic features, DelPhi was utilized to calculate the electrostatic potential on surfaces of the S protein trimer (full structure) and hACE2 RBD. The electrostatic potential distribution on SARS-CoV S protein trimer structure is showed in Figure 2B,E,H and Video S1, which were rendered by Chimera with a color scale from −1.0 to 1.0 kT/e. The charge distribution on SARS-CoV-2 S protein trimer structure is shown in Figure 2C,F,I and Video S2, which were rendered by Chimera with a color scale from −1.0 to 1.0 kT/e as well, for comparison. Negatively and positively charged areas are colored in red and blue, respectively.

By comparing the electrostatic potential on surfaces of two trimer structures, it is obvious that the charge distribution of SARS-CoV and SARS-CoV-2 S proteins are different. From the top view (Figure 2A–C) and the bottom view (Figure 2G–I), we noticed that SARS-CoV has slightly more positively charged area (blue), compared to SARS-CoV-2. It indicates that the SARS-CoV may attract the hACE2 more easily, since the hACE2 binding interface is overall negatively charged (Video S3). Such finding supports the previous studies of our research group [26,27]. The electrostatic distribution differences observed from front views (Figure 2D–F) of the S proteins demonstrate that the electrostatic features may have impacts on the stabilities of the trimers. Here, several details were not investigated about the binding stabilities among monomers in an S protein due to the scope of this work, which mainly focusses on the binding between S protein and hACE2. The electrostatic distributions of S protein RBDs show that the SARS-CoV RBD is more positive, which is consistent with the top view (Figure 2B,C). The bottom of SARS-CoV (Figure 2E,H) has more positive potential than SARS-CoV-2 (Figure 2F,I).

### 2.3. Electric Filed Lines

**Figure 3:**
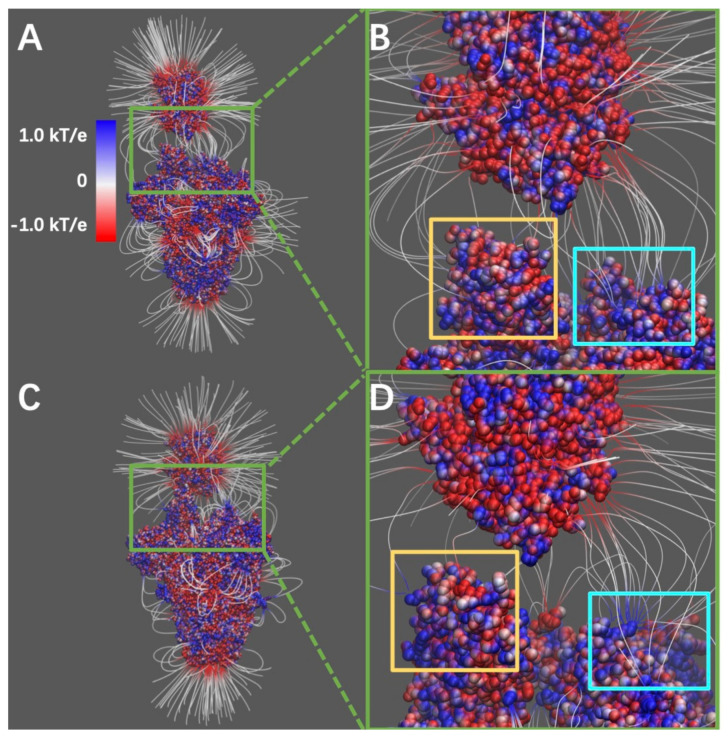
Electrostatic filed lines at the interfaces of S protein and hACE2. (**A**) Electrostatic filed lines between SARS-CoV S protein and hACE2; (**B**) A closeup view of binding domain between SARS-CoV S protein and hACE2; (**C**) electrostatic field lines between SARS-CoV-2 S protein and hACE2; (**D**) A closeup view of binding domain between SARS-CoV-2 S protein and hACE2. Negatively and positively charged areas are colored in red and blue, respectively. Color scale is −1.0 to 1.0 kT/e. Yellow square areas are the RBD of S proteins at open state to reach the hACE2, cyan square areas are the RBD of S proteins at closed state.

Electric field lines surrounding two complex structures were calculated. To better visualize the field lines between interfaces, S protein RBDs are separated from hACE2 RBDs by 10Å (Figure 3). The field line distributions confirmed that both the SARS-CoV and SARS-CoV-2 S protein RBDs have attractive forces to hACE2 protein. In the analysis of field lines, the density of field lines indicates the strength of binding forces, which means that the denser area has the stronger binding interactions. The electric field lines demonstrate that, when hACE2 is away from the S protein, all the three S protein monomers provide attractive interactions to the hACE2. This is expected because the S protein RBDs are positively charged while the hACE2 is negatively charged, as shown in Figure 2 and Video S3, respectively. When hACE2 binds to S proteins (as shown in Figure 1), the hACE2 only binds with one S protein RBD, which is in open state.

Combining the information from Figure 1 and Figure 3, they demonstrate that all the three S protein RBDs generate attractive forces to hACE2. However, when hACE2 becomes closer to an S protein, one S protein RBD flips out and binds to the hACE2 tightly, while the other two S protein RBDs stay in closed state. Even though the monomer with flipped-out S protein RBD is the closest to hACE2 and forms most of the salt bridges and hydrogen bonds, the other two monomers also provide dense field lines and show strong attractive interactions between S proteins and hACE2.

### 2.4. pH-Dependence of Relative Folding Energies

**Figure 4:**
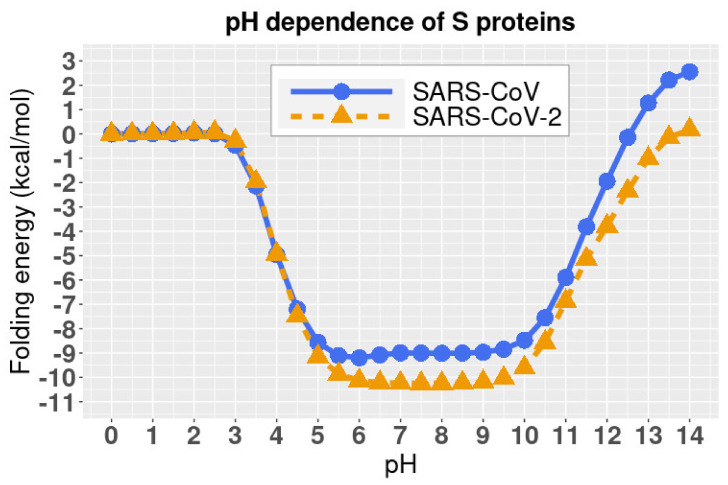
pH dependence of the relative folding energy of S protein RBDs of SARS-CoV and SARS-CoV-2. All values are the differences to the folding energy values at pH = 0, which means that positive values indicate that the energies are higher than the folding energy at pH = 0, and negative values indicate that the energies are lower than the folding energy at pH = 0. So, the larger absolute value is, the larger difference is.

The folding energy of SARS-CoV and SARS-CoV-2 complexes were calculated using DelPhiPKa at different pH values ranging from 0 to 14 with an interval of 0.5 (Figure 4). We observed that SARS-CoV and SARS-CoV-2 have the same trend of folding energy with the change of pH values, which is decreasing from 0 to 6, then becoming stable from 6 to 9, and increasing from 10 to 14. The optimal values locate between 6 to 9 for both of the viruses.

Please note that the folding energies in Figure 4 are relative values because we set the reference energy to be 0 kcal/mol when pH is equal to 0. We did not calculate the absolute values of folding energies since we focused on the pH dependency of the folding energies.

### 2.5. pH-Dependence of Relative Binding Energies

**Figure 5:**
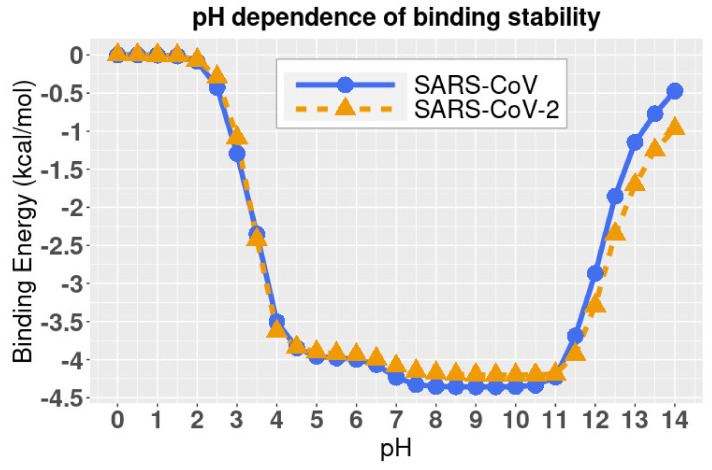
The relative binding energies of complexes at different pH values. All values are the differences to the binding energy values at pH = 0, which means that positive values indicate that the energies are higher than the folding energy at pH = 0, and negative values indicate that the energies are lower than the folding energy at pH = 0. So, the larger absolute value is, the larger difference is.

DelPhiPKa was implemented to calculate the binding energies of two complex structures at different pH values. The results are presented in Figure 5, where we noticed that the binding free energies of both SARS-CoV and SARS-CoV-2 complexes are stable at pH values ranging from 7.5 to 10.5, which indicates that both SARS-CoV and SARS-CoV-2 have a slight preference of weakly basic environment. Please note that the binding energies in Figure 5 are relative values because we set the reference energy to be 0 kJ/mol when pH is equal to 0. We did not calculate the absolute values of binding energies, since we focused on the pH dependency of the binding stability.

Combining the folding energy (Section 2.4) and binding energy (Section 2.5) profiles, we conclude that the best pH environment for both the SARS-CoV and SARS-CoV-2 is from pH 7.5 to 9.

### 2.6. Hydrogen Bonds Analysis

**Figure 6:**
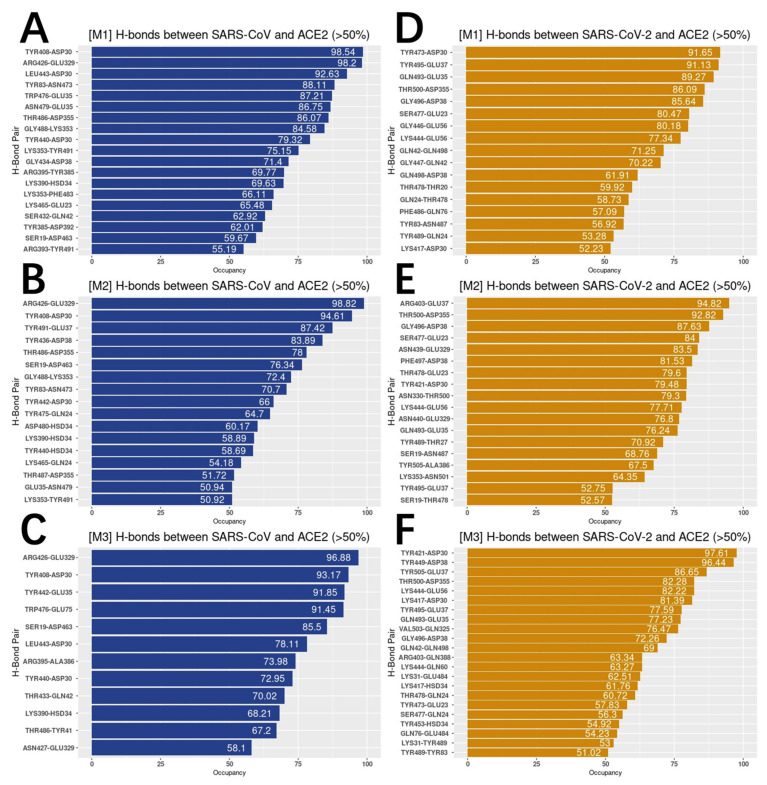
Hydrogen bonds with the frequency above 50%. (**A**–**C**) Hydrogen bonds between SARS-CoV and hACE2 with the frequency above 50% in three independent 100 ns simulations (M1, M2, M3); (**D**–**F**) hydrogen bonds between SARS-CoV-2 and hACE2 with the frequency above 50% in three independent 100 ns simulations (M1, M2, M3).

To analyze the hydrogen bonds distributions on both S proteins RBDs and hACE2 RBD, we got the list of residues forming hydrogen bonds, which are over 50% frequency during the MD simulations (Figure 6).

**Figure 7:**
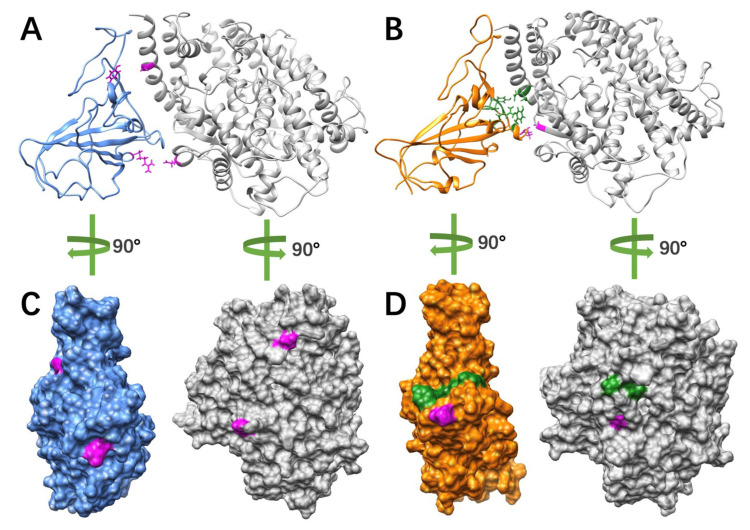
Essential hydrogen bonds distributions at binding interfaces. (**A**) Hydrogen bonds (magenta) on between SARS-CoV RBD (blue) and hACE2 (grey); (**B**) hydrogen bonds (magenta and green) between SARS-CoV-2 RBD (orange) and hACE2 (grey), where magenta colors the essential hydrogen bond pairs found in all three 100 ns simulations, green colors the essential residues that form hydrogen bonds in all three 100 ns simulations but have different pair combinations; (**C**) hydrogen bonds distribution (magenta) on the interface of SARS-CoV RBD (blue) and hACE2 (grey); (**D**) hydrogen bonds distribution (magenta and green) on the interface of SARS-CoV-2 RBD (orange) and hACE2 (grey), where magenta colors the essential hydrogen bond pairs found in all three 100 ns simulations, green colors the essential residues that form hydrogen bonds in all three 100 ns simulations but have different pair combinations. Key residues colored in this figure are involved in forming the top five hydrogen bonds that were found in all three 100 ns simulations for SARS-CoV and SARS-CoV-2 separately.

On average, SARS-CoV forms 16 pairs of hydrogen bonds with over 50% frequency to hACE2, compared to 19 pairs between SARS-CoV-2 and hACE2. For the most essential hydrogen bonds, we colored the top five pairs that were found in all three 100 ns simulations for SARS-CoV and SARS-CoV-2 separately (Figure 7).

From Figure 7, SARS-CoV-2 shows a larger area of hydrogen bond distribution, compared to SARS-CoV-2. When we analyze the key residues that form hydrogen bonds, we noticed that in SARS-CoV-2 complex, two key residues of hACE2 have several acceptors, while SARS-CoV has two stable pairs. The detailed analysis is shown in Table 1.

From Table 1, SARS-CoV prefer sticking to same pairs with strong occupancy while SARS-CoV-2 tends to have a board range of choosing the hydrogen bond acceptors. In the other words, SARS-CoV-2 tends to generate flexible pairs of hydrogen bonds.

## 3. Conclusions

In this work, we applied several computational methods, including MD simulations, DelPhi, and DelPhiPKa, to study the electrostatic features of S proteins for SARS-CoV and SARS-CoV-2. From our results, SARS-CoV and SARS-CoV-2 S protein RBDs both have positively charged interfaces, which provides attractive interactions to hACE2 as hACE2 has negatively charged surface.

Additionally, we revealed the pH-dependence calculations of relative folding energy for SARS-CoV and SARS-CoV-2 S protein RBDs. The best pH to stabilize SARS-CoV and SARS-CoV-2 S protein RBDs is in the range of 6 to 9. The study on pH dependence of binding energies revealed that the complex structures of hACE2 and S proteins of SARS-CoV/SARS-CoV-2 are stable from pH 7.5 to 10.5. Therefore, SARS-CoV and SARS-CoV-2 survive in a similar pH environment. A pH from 7.5 to 9 is the best condition for both SARS-CoV and SARS-CoV-2 to best perform their functions to bind with hACE2.

Furthermore, we analyzed the trajectories from three independent 100ns MD simulations for each complex structure using NAMD and identified essential hydrogen bonds with the involved key residues using VMD. This work mainly focuses on the analyses of hydrogen bonds. Our previous studies discussed ionic interactions in detail. From the MD simulations, we found that SARS-CoV-2 forms an average of 19 pairs of hydrogen bonds with high occupancy (>50%) to hACE2, compared to 16 pairs between SARS-CoV and hACE2. Besides, SARS-CoV tends to stick to same hydrogen bond pairs, while SARS-CoV-2 tends to have a larger range of selections on hydrogen bonds acceptors. We also labelled key residues involved in forming top 5 hydrogen bonds that were found in all three independent 100-ns simulations. This identification is important to potential drug designs for COVID-19 treatments. Our work will shed light on current and future coronaviruses-caused diseases.

## 4. Methods

### 4.1. Structure Preparation

The complex structures of SARS-CoV/hACE2 and SARS-CoV-2/hACE2 were downloaded from the Protein Data Bank (PDB ID 6ACG [32] and 7AD1 [33], respectively). Please note that, in 7AD1, the mutations that the authors made during their experiments are not on the interface area. Since we only focus on the interface area between S proteins and hACE2, the mutations do not affect our results. In this work, we used the complex structures to study the electrostatic binding interactions and the relative binding energies in different pH environments between S proteins and hACE2 RBDs. For the missing loops in proteins, we used MODELLER [34] to model the structures based on the sequences. To understand the mechanisms of S protein binding to hACE2 at the interface particularly, S protein RBDs were separated from the hACE2 binding domain by a distance of 10Å for the better results and visualization. Figure 1 shows the SARS-CoV S protein structure.

### 4.2. Electrostatic Potential Calculation

To study the electrostatic features, DelPhi [35,36] was utilized to calculate the electrostatic potential for the S proteins and hACE2 RBDs. In the framework of continuum electrostatics, DelPhi calculates the electrostatic potential ϕ (in systems comprised of biological macromolecules and water in the presence of mobile ions) by solving the Poisson–Boltzmann equation (PBE):

(1) $$\begin{array}{c} \nabla \cdot \left[\epsilon \left(r\right)\nabla \phi \left(r\right)\right]=-4\pi \rho \left(r\right)+\epsilon \left(r\right)\kappa^{2}\left(r\right)\sinh\left(\phi \left(r\right)/k_{B}T\right) \end{array}$$

where $\;\phi \left(r\right)$ is the electrostatic potential, $\epsilon \left(r\right)$ is the dielectric distribution, $\rho \left(r\right)$ is the charge density based on the atomic structures, κ is the Debye–Huckel parameter, $k_{B}$ is the Boltzmann constant, and T is the temperature. Due to the irregular shape of macromolecules, DelPhi uses a finite difference (FD) method to solve the PBE.

For the preparation of DelPhi calculations, the PQR file of each trimer was generated by PDB2PQR [37]. We used an AMBER [38] force field for PDB2PQR calculation and removed water molecules. For better results, we ensured the new atoms were not rebuilt too close to existing atoms and optimized the hydrogen bonding network.

During DelPhi calculations, the resolution was set as 0.5 grids/Å. The dielectric constants were set as 2.0 for protein and 80.0 for the water environment, respectively. The pH value for the solvent environment was set to be 7.0. The probe radius for generating the molecular surface was 1.4 Å. Salt concentration was set as 0.15 M. The boundary condition for the Poisson Boltzmann equation was set as a dipolar boundary condition. The calculated electrostatic potential on the surface was visualized with Chimera (Figure 2). VMD was used to illustrate electric field lines between S protein and hACE2 (Figure 3). The color scale range was set to be from −1.0 to 1.0 kT/e for the best visual presentation.

### 4.3. Relative Folding Energy Calculation

We used DelPhiPKa [39,40] to calculate pKa values of DNA and UDG, given pH ranging from 0 to 14 with interval of 0.5. During the calculations, we used AMBER force field, and removed water molecules and HETATM. For the hydrogen of ASP and GLU attached atom, we used OD1 and OE1, respectively. The variance of Gaussian distribution was set to be 0.7, salt concentration was 0.15, reference dielectric was 8.0, and external dielectric was 80.0.

The net charges of proteins at the unfolded state were calculated using this equation:

(2) $$Q_{u}\left(pH\right)=\overset{N}{\underset{i=1}{\sum }}\frac{10^{-2.3y\left(i\right)\left(pH-pKa\left(i\right)\right)}}{1+10^{-2.3y\left(i\right)\left(pH-pKa\left(i\right)\right)}}$$

where the summation is of all the titratable groups, and *y*(*i*) value is −1 for acidic groups and +1 for basic groups, respectively. Charge–charge interactions between residues at the unfolded state cause shifts in pKa from values of model compounds [41]. pKa shifts, in turn, affect pH dependence of folding stability, which is governed by this equation [42]:

(3) $$\Delta G\left(\mathrm{pH}\right)=RT\ln10\int_{{\mathrm{pH}}_{0}}^{\mathrm{pH}}(Q_{f}-Q_{u})d\mathrm{pH}\;$$

where $Q_{f}\;$ and $Q_{u}\;$ stand for the net charge of folded and unfolded state, respectively. *R* is the universal gas constant, taken as $1.9872\times 10^{-3}\frac{kcal}{Mol*K}$. *T* is the temperature, with a value of 300 K.

Please note that the algorithms we applied to calculate the folding energies are for the relative values, that is, at pH = 0, the folding energy is 0, and at any other pH values, the folding energies are the relative values to the pH = 0 condition. In Figure 4, all values are the differences to the folding energy value at pH = 0, which means that positive values indicate that the energies are higher than the folding energy at pH = 0, and negative values indicate that the energies are lower than the folding energy at pH = 0. So, the larger absolute value is, the larger difference is.

### 4.4. Relative Binding Energy Calculation

For the binding energy calculation, we involved two methods, which are DelPhiPKa and MM/PBSA [43]. To calculate binding energy using DelPhiPKa, the following equation was used:

(4) $$\Delta N\left(\mathrm{pH}\right)=RT\ln10\int_{{\mathrm{pH}}_{0}}^{\mathrm{pH}}(Q_{t}-Q_{v}-Q_{r})d\mathrm{pH}\;$$

where $\Delta N\left(\mathrm{pH}\right)$ is the binding free energy at different pH values,$\;Q_{t}$ is the total net charges of complex structures (SARS-CoV/SARS-CoV-2 S protein RBD and ACE2 RBD), $Q_{v}$ is the net charges of SARS-CoV/SARS-CoV-2 S protein RBD, and $Q_{r}$ is the net charges of receptor (ACE2 RBD). *R* is the universal gas constant taken as $1.9872\times 10^{-3}\frac{kcal}{Mol*K}$. *T* is the temperature with a value of 300 K.

Please note that the algorithms we applied to calculate the binding energies are for the relative values, that is, at pH = 0 the binding energy is 0 and at any other pH values the binding energies are the relative values to the pH = 0 condition. In Figure 5, all values are the differences to the binding energy value at pH = 0, which means that positive values indicate that the energies are higher than the binding energy at pH = 0, and negative values indicate that the energies are lower than the binding energy at pH = 0. So, the larger absolute value is, the larger difference is.

### 4.5. Molecular Dynamic (MD) Simulations

To simulate the dynamic interactions between S proteins RBD and hACE2 protein, MD simulations [15] were carried out using NAMD [30] with the help of GPUs on Lonestar5 clusters at the Texas Advanced Computing Center (TACC https://www.tacc.utexas.edu/ accessed on 31 March 2021). A 20,000-step minimization was performed for each simulation, followed by 100 million steps, during which 20,000 frames were saved from 100 ns simulations of both SARS-CoV and SARS-CoV-2 separately (1.0 fs per step, 1 frame at each 5000 steps, 100 million steps in total). During the MD simulations, we used CHARMM [44] force field, the temperature was set to 300 K, and the pressure was set to standard using Langevin dynamics. We set the solvent temperature to 300K and solvent pH value to 7 in the MD simulations. In those two simulations, atoms that are not located in binding domains were constrained within a margin of 10.0 Å of their natural movement maximum length values. In order to obtain a more accurate result of the simulation, data of the last 50 ns of each simulation were selected and used for data analysis, since the structure of the first 50 ns is not as stable as the last 50 ns of simulations. The simulation processes are visualized in Videos S4 and S5, generated by VMD. Three independent 100ns MD simulations for each complex structure were performed in this work.

To analyze the interaction between S proteins and hACE2, the hydrogen bonds that formed within the distance of 4Å were extracted from the last 10,000 frames (50 ns) of MD simulations.

## Figures and Tables

**Table 1:** Key residues involved in forming top 5 hydrogen bonds that were found in all three independent 100-ns simulations. Note that SARS-CoV has two same pairs in three simulations, while SARS-CoV-2 has one same pair and two essential residues (GLU37 and ASP38) that form several different pairs in three simulations.

	SARS-CoV		SARS-CoV-2	
Avg. Amount	16		19	
Key residues	Virus	hACE2	Virus	hACE2
	TYR408	ASP30	THR500	ASP355
			TYR495	GLU37
			ARG403	
	ARG426	GLU329	TYR505	
			GLY496	ASP38
			TYR449	

## Data Availability

Not applicable.

## Supplementary Materials

The following supporting information can be downloaded at: https://www.mdpi.com/article/10.3390/pathogens11020238/s1, Figure S1: RMSD comparison of SARS-CoV/ACE2 and SARS-CoV-2/ACE2 complex structure, Figure S2: (A,B) The binding forces of SARS-CoV/hACE2 and SARS-CoV-2/hACE2 complexes. (C–H) X,Y,Z components of electrostatic binding forces. The mass center line was set as the *x* axis. Video S1: SARS-CoV electrostatic surface, Video S2: SARS-CoV-2 electrostatic surface, Video S3: hACE2 electrostatic surface, Video S4: SARS-CoV RBD/hACE2 complex simulation, Video S5: SARS-CoV-2 RBD/hACE2 complex simulation
