# Supplementary material for: The pH Effects on SARS-CoV and SARS-CoV-2 Spike Proteins in the Process of Binding to hACE2
Source: Pathogens. 2022 Feb 11;11(2):238. doi: 10.3390/pathogens11020238 (PMC8879864; doi:10.3390/pathogens11020238)
Supplement: Supplementary file 1 [file pathogens-11-00238-s001.zip › Supplementary.pdf]

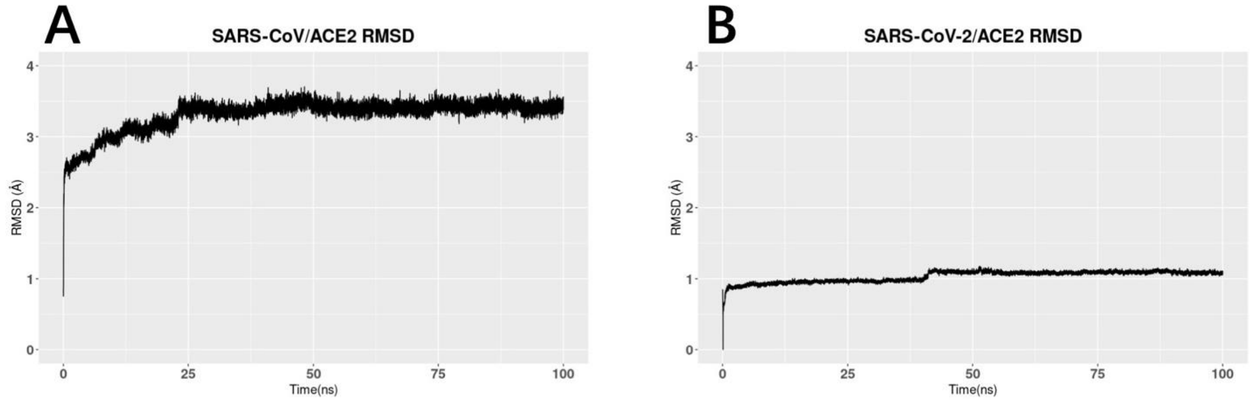

Figure S1. RMSD comparison of SARS-CoV/ACE2 and SARS-CoV-2/ACE2 complex structure

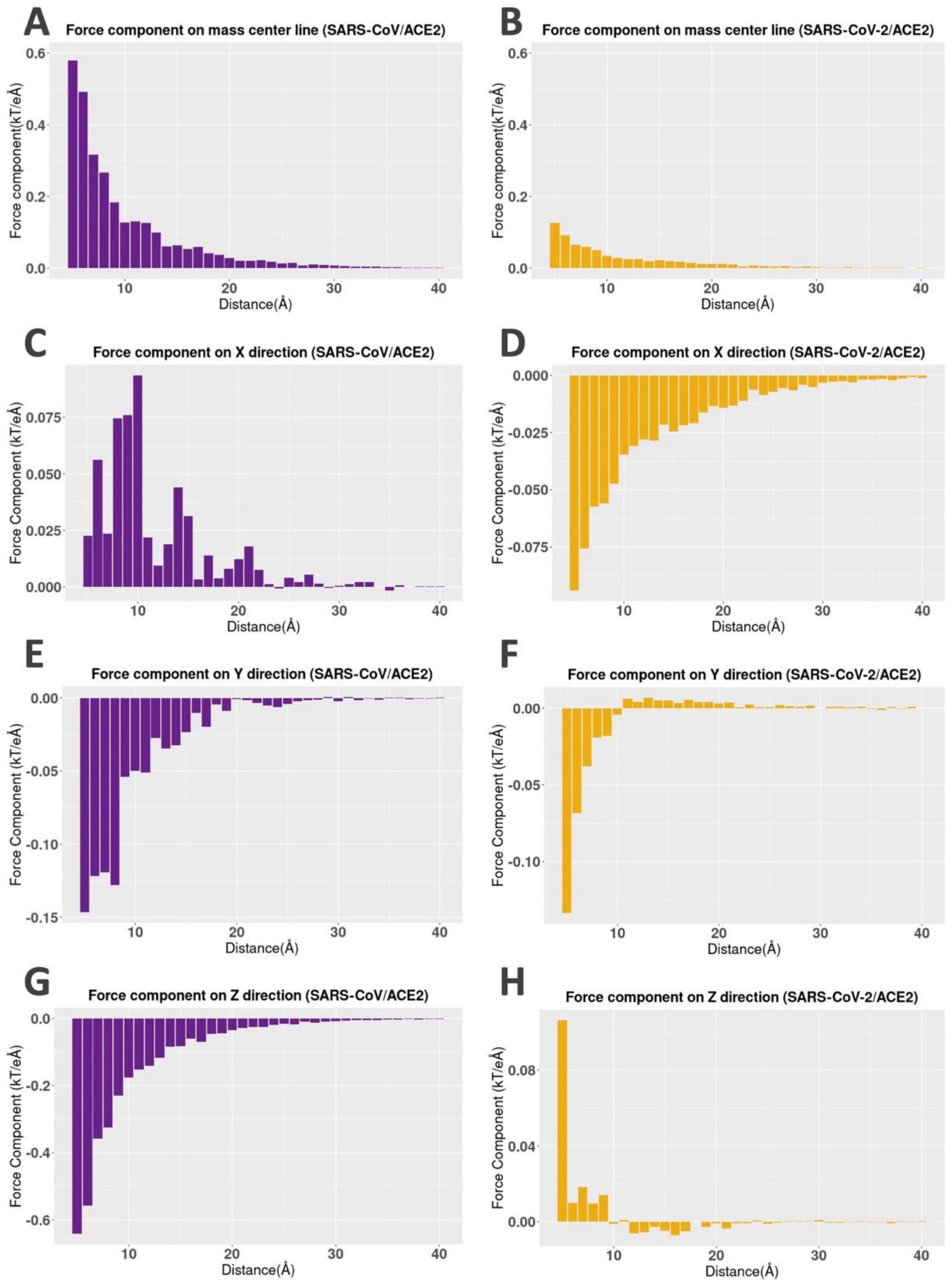

Figure S2. (A–B) The binding forces of SARS-CoV/hACE2 and SARS-CoV-2/hACE2 complexes (C–H) X,Y,Z components of electrostatic binding forces. The mass center line was set as the X axis.

Videos:

Video S1: SARS-CoV electrostatic surface

Video S2: SARS-CoV-2 electrostatic surface

Video S3: hACE2 electrostatic surface

Video S4: SARS-CoV RBD / hACE2 complex simulation

Video S5: SARS-CoV-2 RBD / hACE2 complex simulation

Software applied:

Delphi:

http://compbio.clemson.edu/delphi

DelphiForce:

http://compbio.clemson.edu/delphi-force/

DelphiPKa:

http://compbio.clemson.edu/pka_webserver/

NAMD:

https://www.ks.uiuc.edu/Research/namd/

R Studio:

https://www.rstudio.com/

Chimera:

https://www.cgl.ucsf.edu/chimera/
